# The effects of cage on endplate collapse after stand-alone OLIF: based on finite element analysis and *in vitro* mechanics experiments

**DOI:** 10.3389/fbioe.2024.1508385

**Published:** 2024-12-10

**Authors:** Hao Li, Jiarui Liu, Huifei Cui, Nana Shen, Futong Wu, Zhihao Zhang, Zhongze Zhu, Chensheng Qiu, Hongfei Xiang

**Affiliations:** ^1^ Department of Spine Surgery, The Affiliated Hospital of Qingdao University, Qindao, China; ^2^ Department of Spinal Surgery, Zibo First Hospital, Zibo, China; ^3^ The Department of Rehabilitation Medicine, Affiliated Hospital of Qingdao University, Qingdao, China; ^4^ Department of Spinal Surgery, Qingdao Municipal Hospital, Qingdao, China

**Keywords:** stand-alone OLIF, CAGE, endplate collapse, biomechanics, finite element analysis

## Abstract

**Background:**

Lumbar degenerative diseases are an important factor in disability worldwide, and they are also common among the elderly population. Stand-Alone Oblique Lumbar Interbody Fusion (Stand-Alone OLIF) is a novel surgical approach for treating lumbar degenerative diseases. However, long-term follow-up after surgery has revealed the risk of endplate collapse associated with Stand-Alone OLIF procedures. This study aimed to investigate the effect of the cage factor on endplate collapse after Stand-Alone OLIF.

**Methods:**

Finite element (FE) models and calf lumbar functional units were established separately and used to simulate Stand-Alone OLIF surgery. On the L5 endplate of the FE model and the calf lumbar functional unit, 12 cage positions from anterior to posterior, 16 cage inclination angles from 0° to 15°, and 4 cage heights were selected to simulate surgical models with different cage positions. Compression loads of 400N were applied to the upper surface of the superior vertebral body of the cage, and 10Nm torques in four directions were used to simulate four different physiological movements of the lumbar spine: flexion, extension, lateral curvature and torsion, in order to compare the range of motion of the surgical segment and the endplate stress.

**Results:**

When the cage is placed closer to the anterior and posterior edges of the endplate and when the height of the cage exceeds 12mm, the intervertebral range of motion at the surgical segment is greater and the stress on the endplate is higher during various lumbar spine activities. When the cage is inclined at an angle within 15°, there are no significant differences in the corresponding endplate stress and the range of motion.

**Conclusion:**

For Stand-Alone OLIF surgery, inserting the cage in the central anterior-posterior position of the intervertebral space and selecting a cage with a height not exceeding 12 mm can reduce the stress on the endplate after surgery, which is more conducive to the stability of the lumbar spine postoperatively and reduces the risk of postoperative endplate collapse. The inclination angle of the cage placement does not significantly affect postoperative endplate stress or lumbar stability.

## 1 Introduction

Lumbar degenerative diseases are are a common condition among the elderly and are a significant cause of disability worldwide. Lumbar degenerative diseases, including lumbar spondylolisthesis, intervertebral disc degeneration, and lumbar spinal stenosis, cause symptoms such as mechanical low back pain, radiculopathy pain, and claudication, which can severely affect the patient’s mobility and quality of life (Ravindra et al., 2018; Mobbs et al., 2015). When conservative treatment fails, lumbar interbody fusion is a common surgical procedure that can provide decompression, maintain lumbar stability, and correct deformities (Ravindra et al., 2018). After nearly 100 years of development, there is a wide variety of lumbar fusion approaches, such as Posterior Lumbar Interbody Fusion (PLIF), Anterior Lumbar Interbody Fusion (ALIF), Transforaminal Lumbar Interbody Fusion (TLIF), and Lateral Lumbar Interbody Fusion (LLIF) (Rathbone et al., 2023; Meng et al., 2021). Although the above surgical methods can achieve good clinical results, they are constrained by the surgical approach. In PLIF, the dura mater and nerve roots need to be retracted, TLIF may potentially damage the nerve root exit zone, ALIF is prone to damaging abdominal blood vessels and visceral nerves, and LLIF may cause damage to important structures and nerves such as the psoas major muscle and lumbar plexus (Mobbs et al., 2015; Lan et al., 2018; Verma et al., 2020).

The Oblique Lumbar Interbody Fusion (OLIF), first reported by Silvestre in 2012, is a novel procedure for lumbar interbody fusion (Li et al., 2020). Compared to traditional lumbar fusion surgeries, OLIF establishes a working channel through the anatomical space between the abdominal aorta and the psoas major muscle to access the intervertebral disc space without disrupting the posterior spinal column structure. This approach avoids direct injury to the paraspinal tissues, spinal canal, and nerves, and allows for the insertion of larger interbody cages. It provides distraction for the foraminal decompression and endplate, facilitating rapid and thorough fusion (Phan et al., 2016). Applying the Stand-Alone technique to OLIF surgery eliminates the need for additional internal fixation systems, making it more minimally invasive, reducing the patient’s economic burden and surgical trauma. Clinical follow-up studies have demonstrated that Stand-Alone OLIF can achieve similar therapeutic effects to OLIF combined with posterior pedicle screw fixation systems (Huo et al., 2020; He et al., 2020a; He et al., 2020b).

OLIF can achieve indirect decompression by restoring the height of the intervertebral disc space, thereby increasing the area of the spinal canal and intervertebral foramina, so the subsidence of the fusion device is one of the complications that should not be overlooked after surgery (Ran et al., 2024). Endplate subsidence not only can lead to a reduction in the height of the intervertebral space at the surgical segment, ligamentous laxity, loss of stability, and fusion failure,but it may also result in spinal canal or foraminal stenosis, compression of the spinal cord or nerves, recurrence of low back and leg pain, and in severe cases, may even necessitate revision surgery. Thus, early recognition and timely intervention of risk factors for endplate subsidence following Stand-Alone OLIF surgery are crucial for enhancing surgical success rates and patient satisfaction after the surgery (Shen et al., 2024). Endplate damage, osteoporosis, lumbar instability, spondylolytic spondolisthesis, grade II or higher lumbar spondolisthesis, and multi-segment fusion are considered high-risk factors for endplate subsidence after Stand-Alone OLIF surgery (Li et al., 2020; Ran et al., 2022; Zhao et al., 2022; Kotheeranurak et al., 2023). For Stand-Alone OLIF, as there is no internal fixation system to distribute the stress, the stress is concentrated entirely on the cage and the adjacent endplates (Wang et al., 2021), which may increase the risk of endplate subsidence after surgery. Previous research has demonstrated a correlation between endplate morphology and the occurrence of endplate subsidence (Hu et al., 2023). It is inferred that the selection of the cage may also exert a certain influence on endplate subsidence in Stand-Alone OLIF, yet there is a lack of systematic research in this area. This experiment will investigate the impact of the anterior-posterior position, inclination angle, and height of the cage on endplate subsidence following Stand-Alone OLIF surgery.

Existing biomechanical data indicates that an intact spinal bone unit is a necessary prerequisite for maintaining the normal physiological function of load-bearing in the spine (Xue et al., 2024). Finite Element Analysis (FEA) is a widely utilized tool across various research fields. With the application of FEA in spinal biomechanics, it helps to better understand the behavior of the spine under physiological and pathological conditions, and facilitates the design and application of spinal devices. When investigating the impact of cage on endplate subsidence after Stand-Alone OLIF surgery, FEA enables a quantitative and intuitive display of changes in lumbar mobility after surgery and the stress conditions on the cage and endplates, guiding clinical physicians in selecting the most appropriate cage for the patient (Wang and Wu, 2023). However, since data obtained solely from FEA lacked sufficient credibility, we conducted *ex vivo* biomechanical experiments using calf lumbar spines to further validate the results of the FEA.

## 2 Materials and methods

### 2.1 Subjects

**FIGURE 1:**
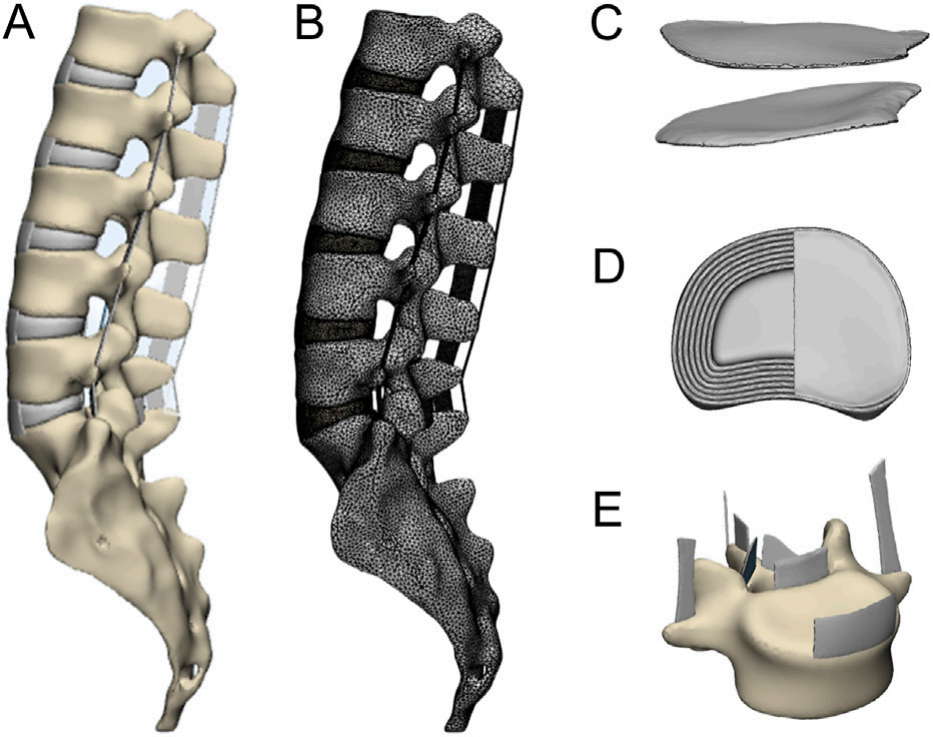
**(A)** Complete lumbar model, **(B)** the lumbar spine model after dividing the grid, **(C)** endplate, **(D)** intervertebral disc, **(E)** ligament.

The lumbar spine model was established using data from a healthy adult male volunteer, as depicted in Figure 1. Based on clinical imaging examinations, the volunteer, aged 24, was 180 cm tall, weighed 62 kg, had no history of spinal disease or injury, and signed an informed consent form. We used a Siemens 64-slice 128-layer spiral CT with a slice thickness of 0.625 mm to scan the volunteer’s L1-S1 segment, capturing 656 DICOM format images. The lumbar spine from an 18-month-old calf, with no lumbar spine abnormalities confirmed by CT scan, was stored in sealed bags at −20°C after processing, to be thawed before use. This study was approved by the hospital’s review committee and the ethics committee.

### 2.2 Construction of lumbar finite element (FE) model

We imported the 656 DICOM-formatted CT data into Mimics, used the “CT BONE” tool to individually select the L1-S1 vertebrae from the 2D images, selected the grayscale values suitable for bone tissue, and reconstructed the 3D models of each vertebra. For areas where the edges of the vertebrae are unclear, we manually segmented each layer on the 2D images to ensure the integrity of the vertebrae while separating them from adjacent vertebrae. In order to ensure no gaps were present in any layer of the two-dimensional images, we filled any holes in the extracted 3D vertebrae, then we used the “Smooth” tool to properly smooth the vertebrae, saved the resulting vertebral contours in STL format, and imported them into 3-Matic.

As shown in Figure 1, using the positive engineering software 3-matic to extract appropriately sized surfaces on the upper and lower aspects of adjacent vertebrae to construct the endplates and intervertebral discs, setting the endplate thickness to 0.5 mm. The intervertebral disc comprises a central nucleus pulposus and a surrounding annulus fibrosus. We used the “Offset” tool to separate the cortical bone from the cancellous bone, setting the cortical bone thickness to 1 mm. Based on the anatomical structure of the lumbar spine, we respectively established the anterior longitudinal ligament, posterior longitudinal ligament, ligamentum flavum, intertransverse ligament, spinous interligament, supraspinous ligament, and capsular ligament. Lastly, we performed meshing on each component and pre-defined the contact surfaces between the components.

We imported the meshed model into Abaqus, assigned material properties based on previous research (Nishida et al., 2019), and set up two analysis steps and loads. In the first analysis step, a load of 400 N axial compression was applied to the upper surface of L1 to simulate the load of one’s own body weight. While in the second analysis step, a torque of 10 Nm was applied to the upper surface of L1 to simulate movements in the directions of flexion, extension, lateral bending, and torsion. Restrict the movement of S1 in all six degrees of freedom to serve as a fixation, set the friction coefficient of the facet joints to 0.2, and configure the other contact types as a bonded interaction (Table 1).

**TABLE 1:** Material properties of the model.

Component	Modulus of elasticity	Poisson’s ratio
os integumentale	12,000	0.3
cancellous bone	100	0.3
endplate	3000	0.4
rear structure	3500	0.25
nucleus pulposus	1	0.49
annulus fibrosus	4.2	0.45
lacertus medius	20	0.3
ligamenta longitudinale posterius	20	0.3
ligamenta intertransversaria	59	0.3
ligamentum flavum	19.5	0.3
ligamenta interspinalia	12	0.3
ligamenta supraspinale	15	0.3
arthrocystis ligament	32.6	0.3
cage	3600	0.3

### 2.3 Construction of stand-alone OLIF FE model

**FIGURE 2:**
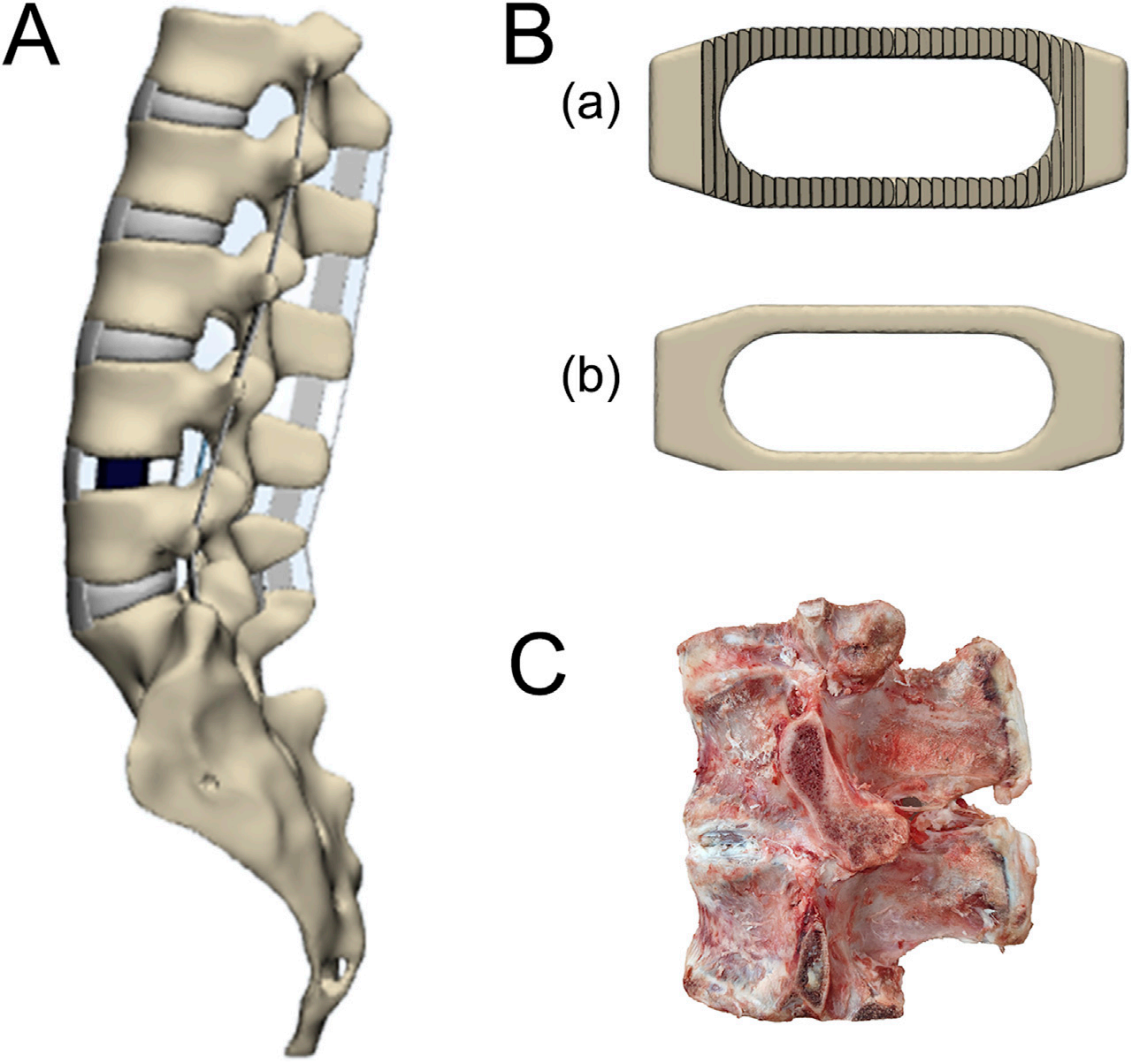
**(A)** The Stand-Alone OLIF surgical model, **(B) (A)** the original cage model, and **(B)** remove the cage mode of the dentate structure, **(C)** calf lumbar spine functional unit.

**FIGURE 3:**
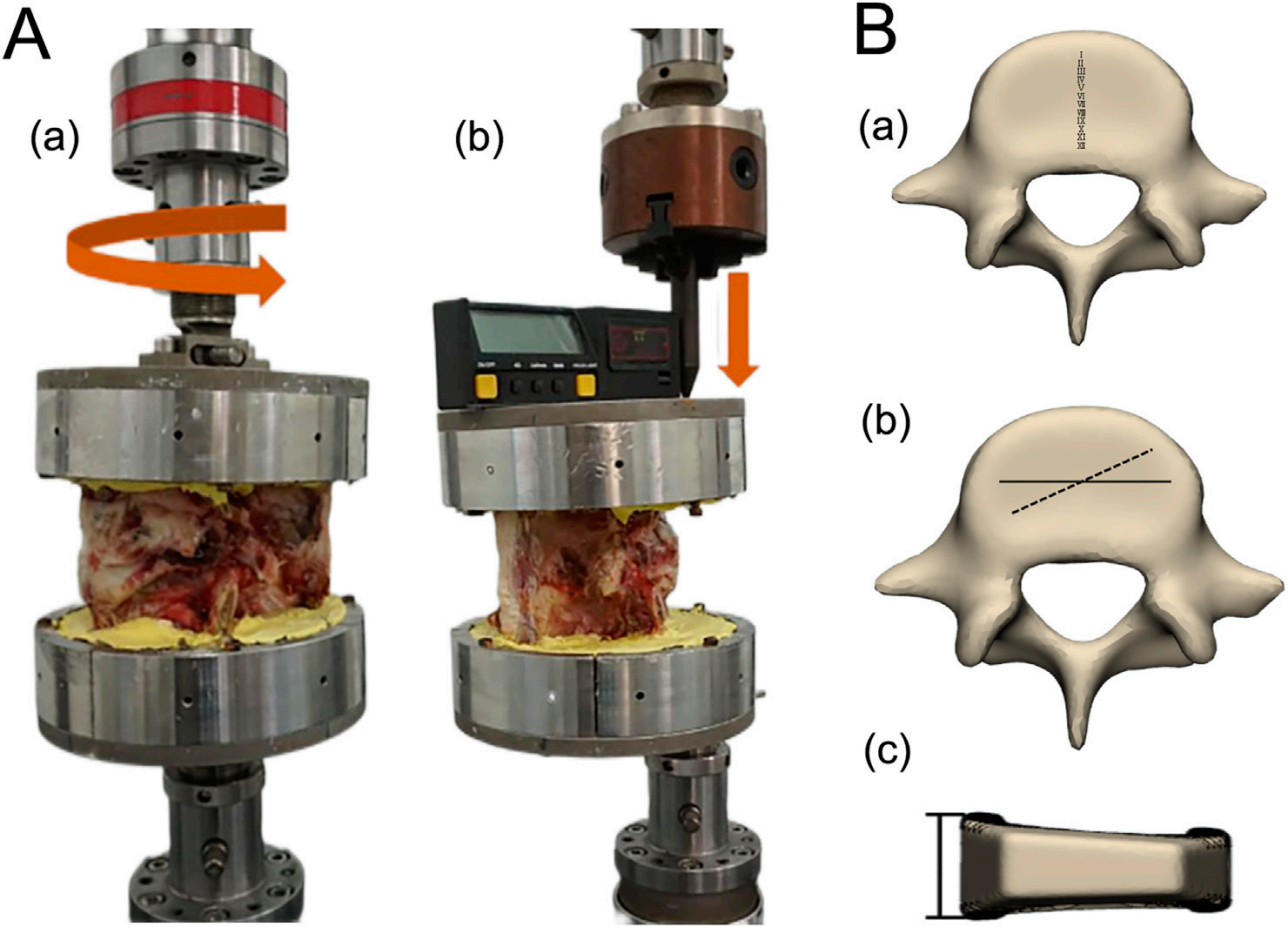
**(A)** *In vitro* mechanical experiments **(A)** flexion, extension, lateral curvature loads, and **(B)** torsion load. **(B)** Schematic diagram of **(A)** anterior-posterior position, **(B)** obliquity and **(C)** height of the cage placement.

Taking L4-L5 as the surgical segment, we simulated the Stand-Alone OLIF by removing the left annulus fibrosus and intervertebral disc at the surgical segment, then implanted the cage (Figure 2A). To avoid excessive stress concentration, we removed the serrated structures on the upper and lower surfaces of the cage (Figure 2B). The implantation position of the cage is defined as the projected position of the center point of the device on the upper endplate of L5. Moro et al. (Moro et al., 2003) divided the area between the anterior and posterior edges of the vertebral body into four regions (Figure 3B). Based on this, we further refined these four regions into 12 distinct areas and established corresponding 12 surgical models (P1-P12). The inclination angle is defined as the angle between the horizontal centerline of the cage and the horizontal centerline of the upper endplate of L5. Sixteen surgical models (A0-A15) were established with the inclination angles of the implanted cage set at 0°–15° increments. Finally, we established four surgical models (H1-H4) with the height of the cage set at 8mm, 10mm, 12mm, and 14 mm respectively.

### 2.4 *Ex vivo* biomechanical experiment

We selected a properly sized calf lumbar spine specimen (Figure 2C), appropriately frozen it, moistened its surface with 0.9% physiological saline, and removed as much excess paravertebral muscle and fat as possible without damaging the vertebrae, considering two adjacent vertebrae, the intervertebral disc, and the surrounding ligaments as a single functional lumbar unit for research. By simulating the Stand-Alone OLIF, we transected the intervertebral disc of the lumbar functional unit, removed the nucleus pulposus and the left annulus fibrosus, appropriately distracted the intervertebral space, and inserted a cage, taking care to avoid damaging the endplates during the procedure. CT scanning is performed to ensure satisfactory positioning of the cage. We designed and customized a fixture for connecting each lumbar functional unit to the mechanical testing machine, then encased each lumbar functional unit in plaster within the fixture, covered it with petroleum jelly, and sealed it for storage in a −20°C refrigerator. The above specimen preparation process refers was based on previous methods for creating calf lumbar spine specimens and specimens for Stand-Alone OLIF surgery (Zhang et al., 2021).

The experimental specimens were randomly divided into three groups: (1) The anterior-posterior position group of the cage, with 12 samples, where 12 identical cages were inserted at 12 positions from anterior to posterior at a 0° tilt angle into the specimens (p1-p12); (2) The tilt angle group of the cage, with 16 samples, where 16 identical cages were inserted at tilt angles ranging from 0°–15° into the central anterior-posterior position of the specimens (a0-a15); (3) The height group of the cage, with 4 samples, where cages with heights of 8mm, 10mm, 12mm, and 14 mm were inserted at a 0° tilt angle into the central anterior-posterior position of the specimens (h1-h4). During the procedure, each specimen was maintained in a moist state to reduce the impact of desiccation on the mechanical properties of the specimen.

Before the experiment, we thawed the specimens, then fixed the specimens in the fixture, mounted the fixture onto the mechanical testing machine, checked the sensors, adjusted the position of the force arm, calibrated and tested for stability. A 10Nm vertical load was applied in three directions, each at a distance of 8.5 cm from the center of the fixture, to simulate flexion, extension, and lateral bending movements, additionally, a 10Nm torque was applied to simulate torsional movement (Figure 3A). Each condition was tested three times with a 30-s interval between tests to eliminate the effects of creep and relaxation, using the third test result as the final data.

### 2.5 Data measurement and processing

Given that endplate subsidence after Stand-Alone OLIF surgery predominantly occurs at the inferior endplate of the upper vertebra (Hou and Luo, 2009), the endplate stress in this study’s FEA and *ex vivo* biomechanical experiments was derived from the inferior endplate of the L4 vertebra. Given that Stand-Alone OLIF involves the resection of the left annulus fibrosus in the intervertebral space, which has a greater impact on physiological activities toward the left side, this study uses left lateral bending and left torsion as representative activities for side bending and torsion. The FE data and *ex vivo* biomechanical experimental data that were collected were processed and analyzed using the statistical software SPSS 22.0. After testing for homogeneity of variance and performing a *t*-test, regression analysis was conducted. A difference was considered to have statistical significance if the *p*-value was less than 0.05.

## 3 Results

### 3.1 FE model validation

**FIGURE 4:**
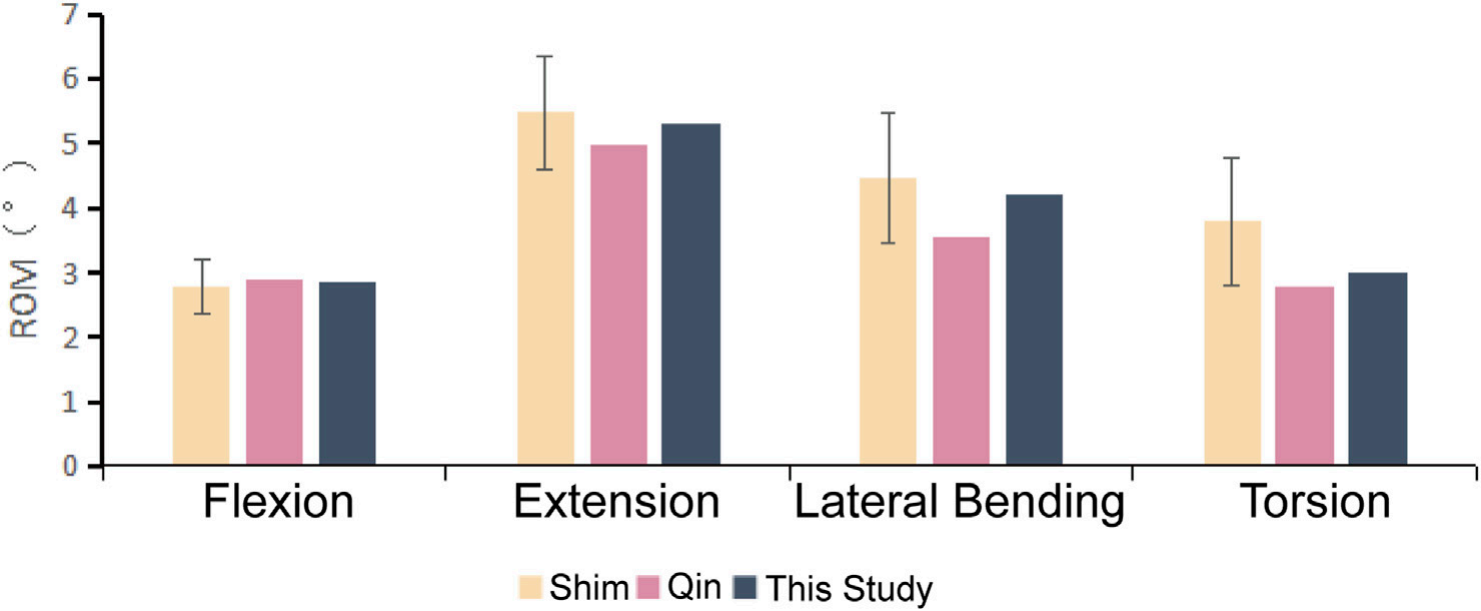
The activity of the L4-L5 segmental mobility in this FE model compared with the literature references.

Upon comparing the range of motion in each direction of the intact FE model L4-L5 segment established in this study with previous *ex vivo* biomechanical experiments (Shim et al., 2008) and finite element studies (Qin et al., 2022), we found the results were similar to those of prior research and all were within one standard deviation (Figure 4). This validates that the FE model developed in this study is suitable for further research analysis.

### 3.2 Impact of the anterior-posterior position of the cage

#### 3.2.1 Comparative analysis of intervertebral ROM

**FIGURE 5:**
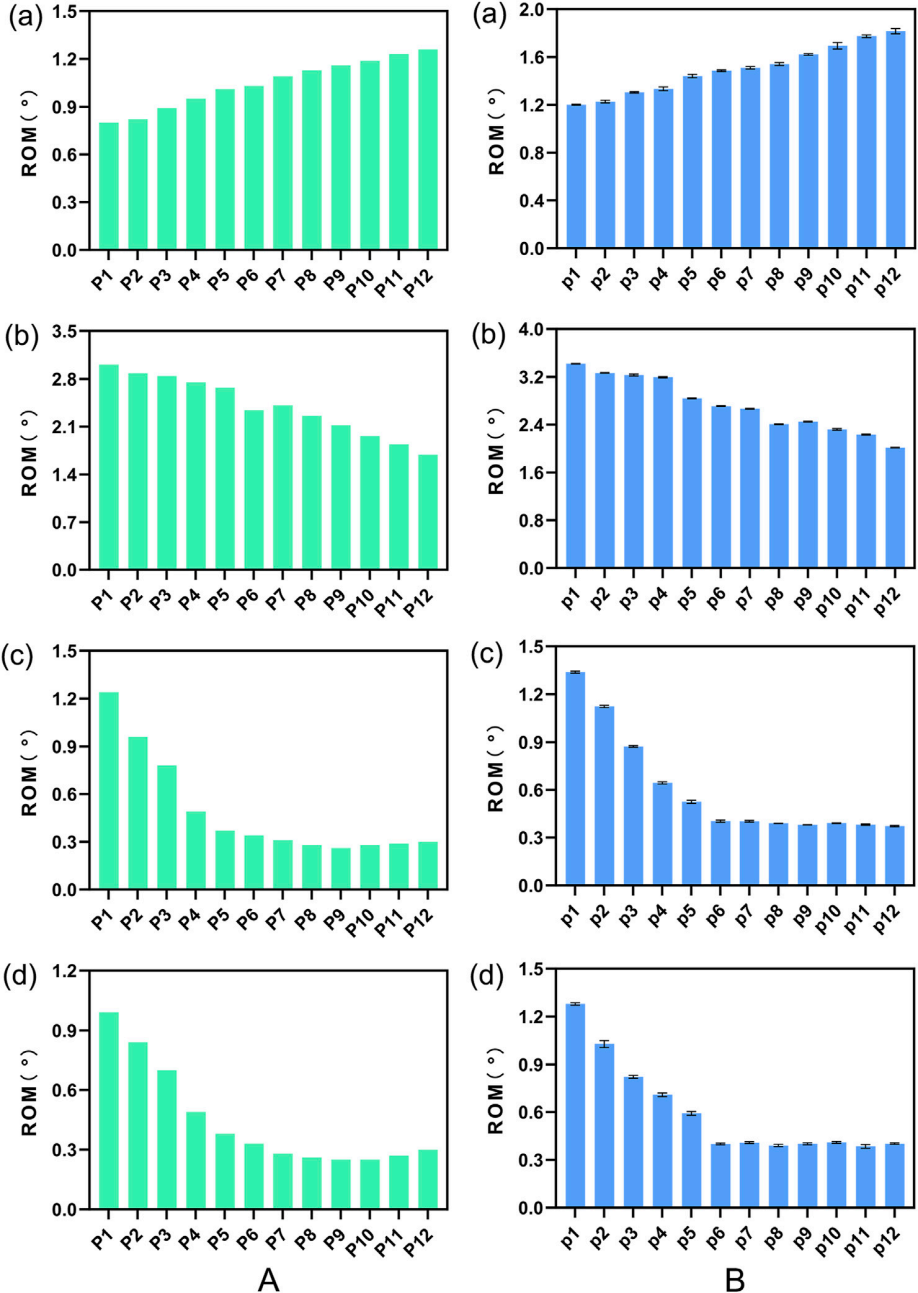
The ROM of **(A)** FE model L4-L5 segment and **(B)** calf lumbar spine model under various physiological activities: **(A)** flexion, **(B)** extension, **(C)** lateral curvature, **(D)** torsion when the cage is positioned at different locations.

Figure 5 illustrates a comparison of the Range of Motion (ROM) at the L4-5 segment under flexion, extension, lateral bending, and torsion for 12 Stand-Alone OLIF FE models (P1-P12) and the corresponding calf lumbar spine models (p1-p12). Figure 5A indicates that the range of motion in each direction of the L4-L5 segment in the FE model is less than that of the intact FE model. Comparing with Figure 4, it demonstrates that the stability of the lumbar spine is increased after Stand-Alone OLIF surgery. During flexion, the closer the cage is to the posterior edge of the vertebral body, the greater the intervertebral range of motion; during extension, lateral bending, and torsion, the closer the cage is to the anterior edge of the vertebral body, the greater the intervertebral range of motion, *p* < 0.05. Greater mobility implies poorer stability. Upon comprehensive analysis, it is concluded that placing the cage in the central position on the endplate anterior-posterior axis yields the best postoperative lumbar stability. As shown in Figure 5B, the trend of mobility changes in the calf lumbar spine model under various conditions with the position of the cage is consistent with that of the FE model, *p* < 0.05, which confirms the reliability of the FEA results.

#### 3.2.2 Comparative analysis of endplate stress

**FIGURE 6:**
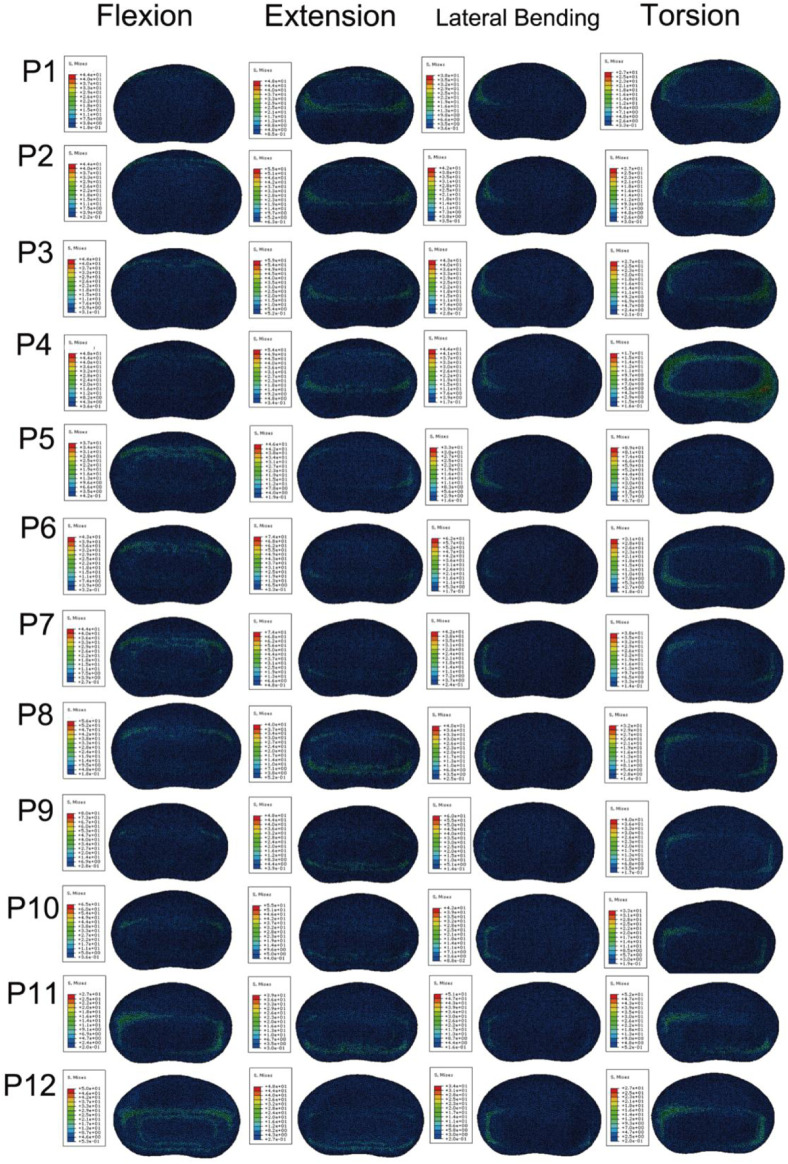
Stress cloud map of the L4 lower endplate in FE models with cages at 12 anterior and posterior positions under various conditions.

**FIGURE 7:**
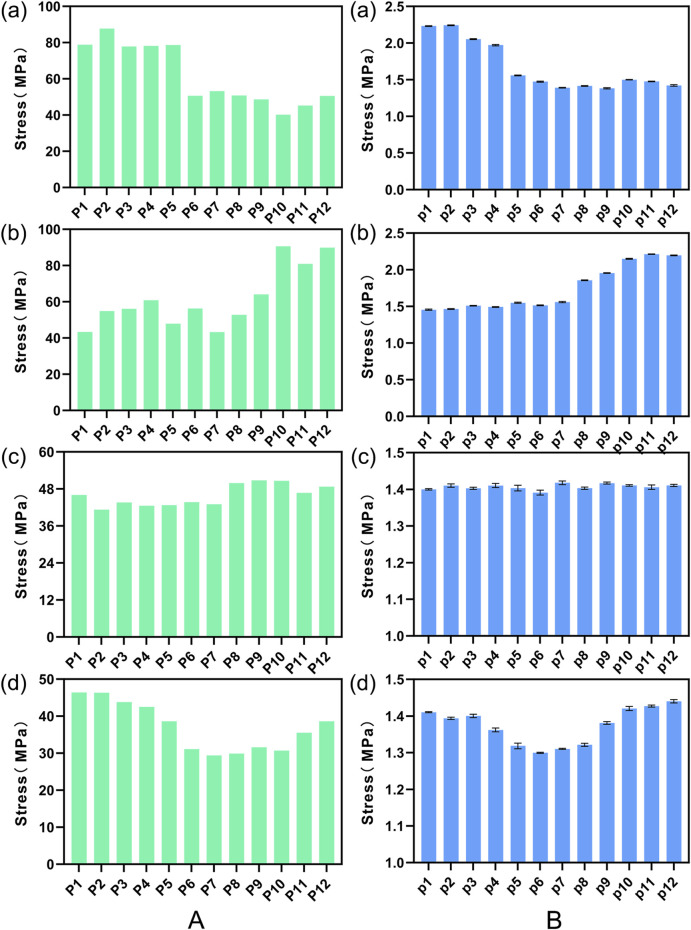
Stress on the L4 lower endplate under various conditions in **(A)** FE models and **(B)** calf lumbar spine models: **(A)** flexion, **(B)** extension, **(C)** lateral curvature, **(D)** torsion when the cage is positioned at different locations.

To more vividly depict the stress conditions of the L4 inferior lamina under different conditions in the FE model, we created a stress contour plot (Figure 6). As shown in Figure 6, in the FE model, when the cage is located at the anterior and posterior edges of the endplate, the stress on the L4 inferior endplate is the greatest during flexion, extension, and torsion, while the stress change is not significant during lateral bending. Figure 7 illustrates a comparison of the stress on the L4 inferior endplate under four conditions: flexion, extension, lateral bending, and torsion for 12 Stand-Alone OLIF FE models (P1-P12) and the corresponding calf lumbar spine models (p1-p12). Figure 7A indicates that in the FE model, during flexion, the closer the cage is to the anterior edge of the endplate, the greater the stress on the L4 inferior endplate; during extension, the closer the cage is to the posterior edge of the endplate, the greater the stress on the L4 inferior endplate, *p* < 0.05; during lateral bending, there is no significant relationship between the position of the cage and the endplate stress, *p* > 0.05; during torsion, the closer the cage is to the anterior or posterior edge of the endplate, the greater the stress on the L4 inferior endplate, *p* < 0.05. Upon comprehensive analysis, when the cage is placed in the central position on the endplate, the stress on the L4 inferior endplate is minimized, and the probability of postoperative endplate subsidence is the lowest. As shown in Figure 7B, the trend of stress on the L4 inferior endplate in various conditions of the calf lumbar spine Stand-Alone OLIF surgical model changes with the position of the cage, which is consistent with the FE model, *p* < 0.05, which further confirms the reliability of the FEA results.

### 3.3 Impact of the insertion angle of the cage

#### 3.3.1 Comparative analysis of intervertebral ROM

**FIGURE 8:**
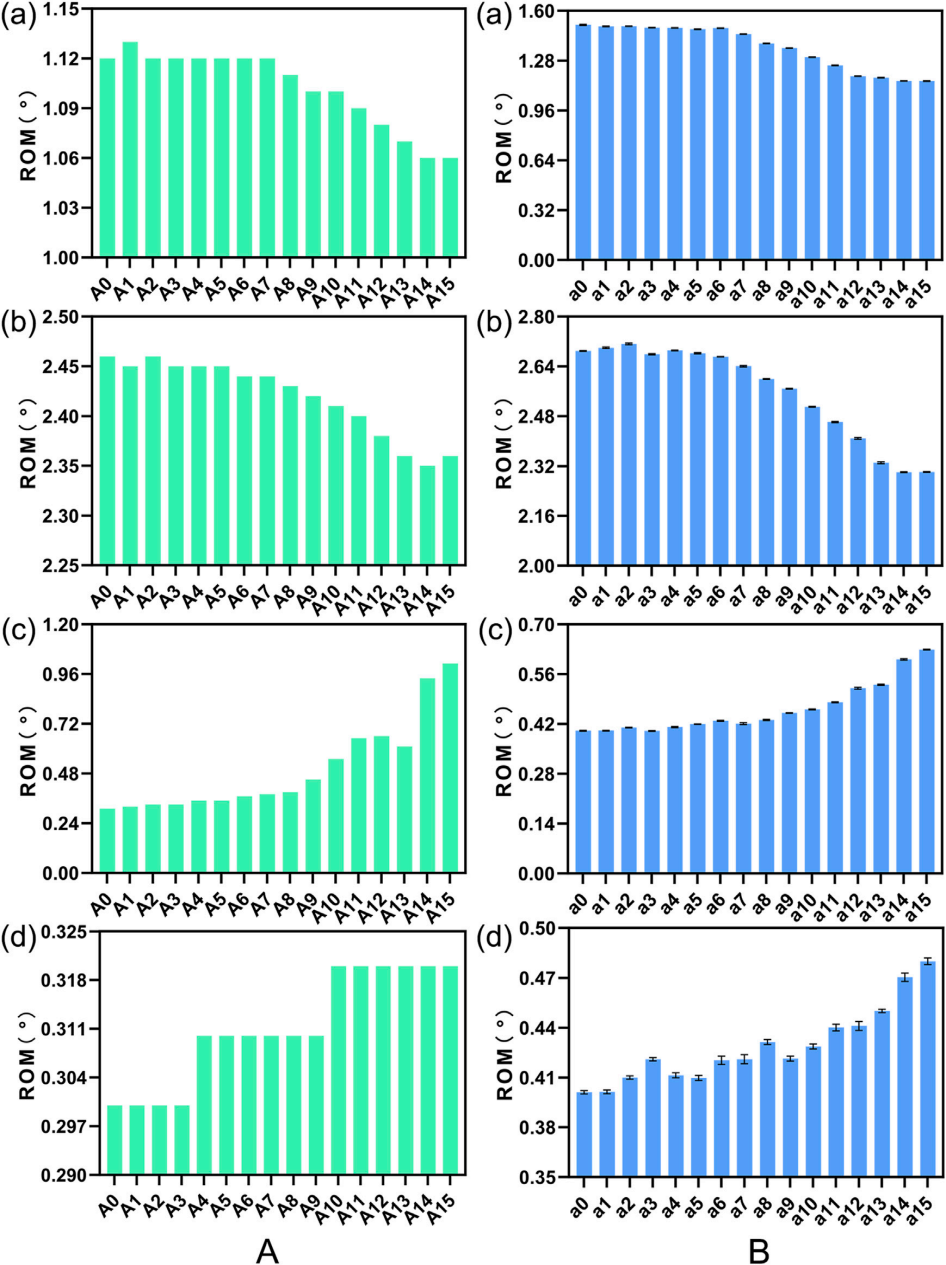
The ROM of **(A)** FE model L4-L5 segment and **(B)** calf lumbar spine model under various physiological activities: **(A)** flexion, **(B)** extension, **(C)** lateral curvature, **(D)** torsion when the cage is positioned at different angles.

Figure 8 illustrates a comparison of the Range of Motion (ROM) at the L4-5 segment under flexion, extension, lateral bending, and torsion for 16 Stand-Alone OLIF FE models (A0-A15) and the corresponding calf lumbar spine models (a0-a15). Figure 8A indicates that the range of motion in each direction of the L4-L5 segment in the FE model is less than that of the intact FE model. Comparing with Figure 4, it demonstrates that the stability of the lumbar spine is increased after Stand-Alone OLIF surgery. During flexion and extension activities, the greater the insertion angle of the cage, the smaller the intervertebral range of motion, *p* < 0.05; during lateral bending and torsional activities, the greater the insertion angle of the cage, the larger the intervertebral range of motion, *p* < 0.05. The greater the intervertebral range of motion, the worse the postoperative stability of the lumbar spine. An excessively large insertion angle of the cage affects the stability of the lumbar spine during lateral bending and torsional activities, while an overly small insertion angle affects the stability of the lumbar spine during flexion and extension activities. As shown in Figure 8B, the trend of intervertebral range of motion changes with the insertion angle of the cage in various conditions of the calf lumbar spine model is consistent with that of the FE model, *p* < 0.05, which confirms the reliability of the FEA results. Under the same conditions, the *ex vivo* experimental range of motion is greater than the results of the FEA.

#### 3.3.2 Comparative analysis of endplate stress

**FIGURE 9:**
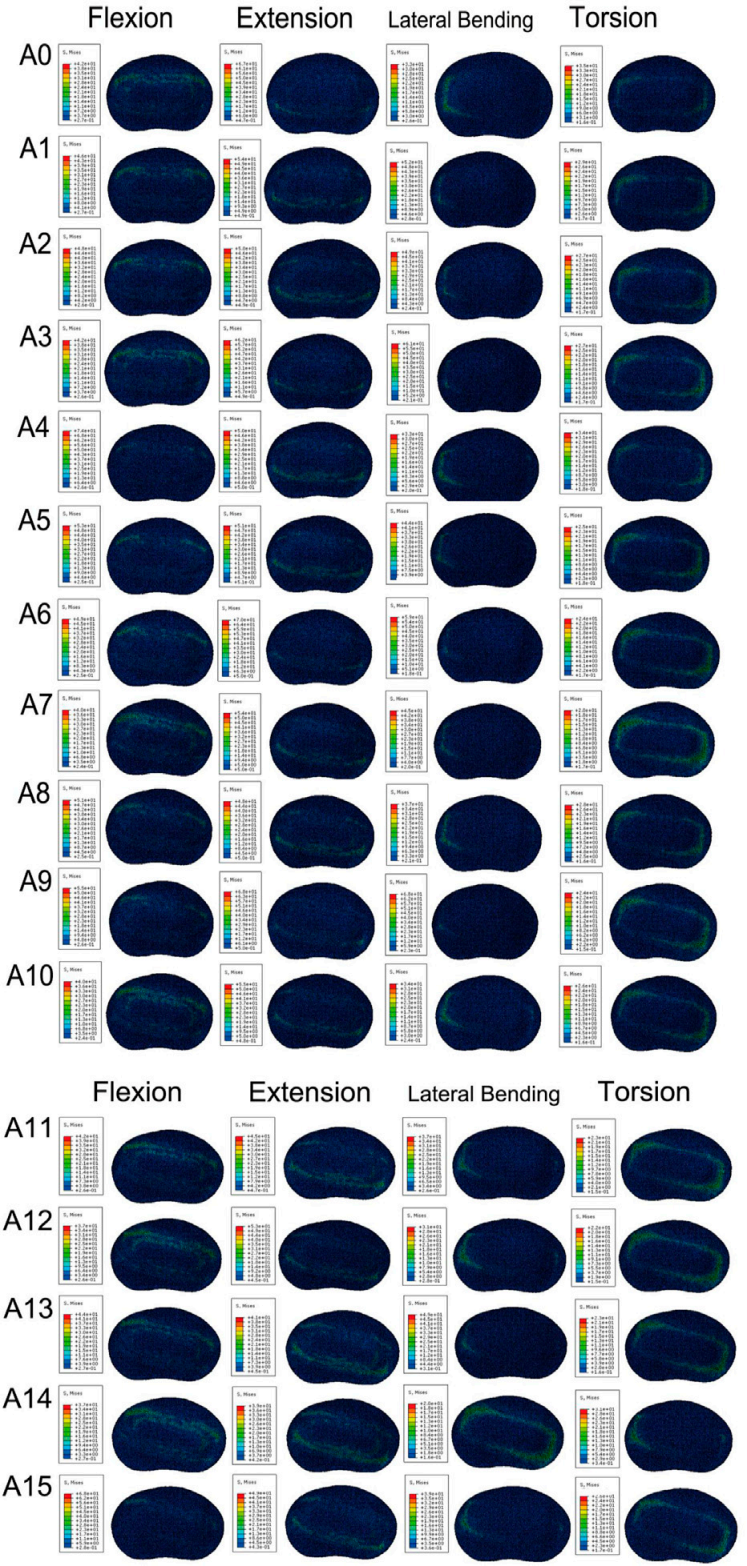
Stress cloud map of the L4 lower endplate in FE models with cages at 16 different angles under various conditions.

**FIGURE 10:**
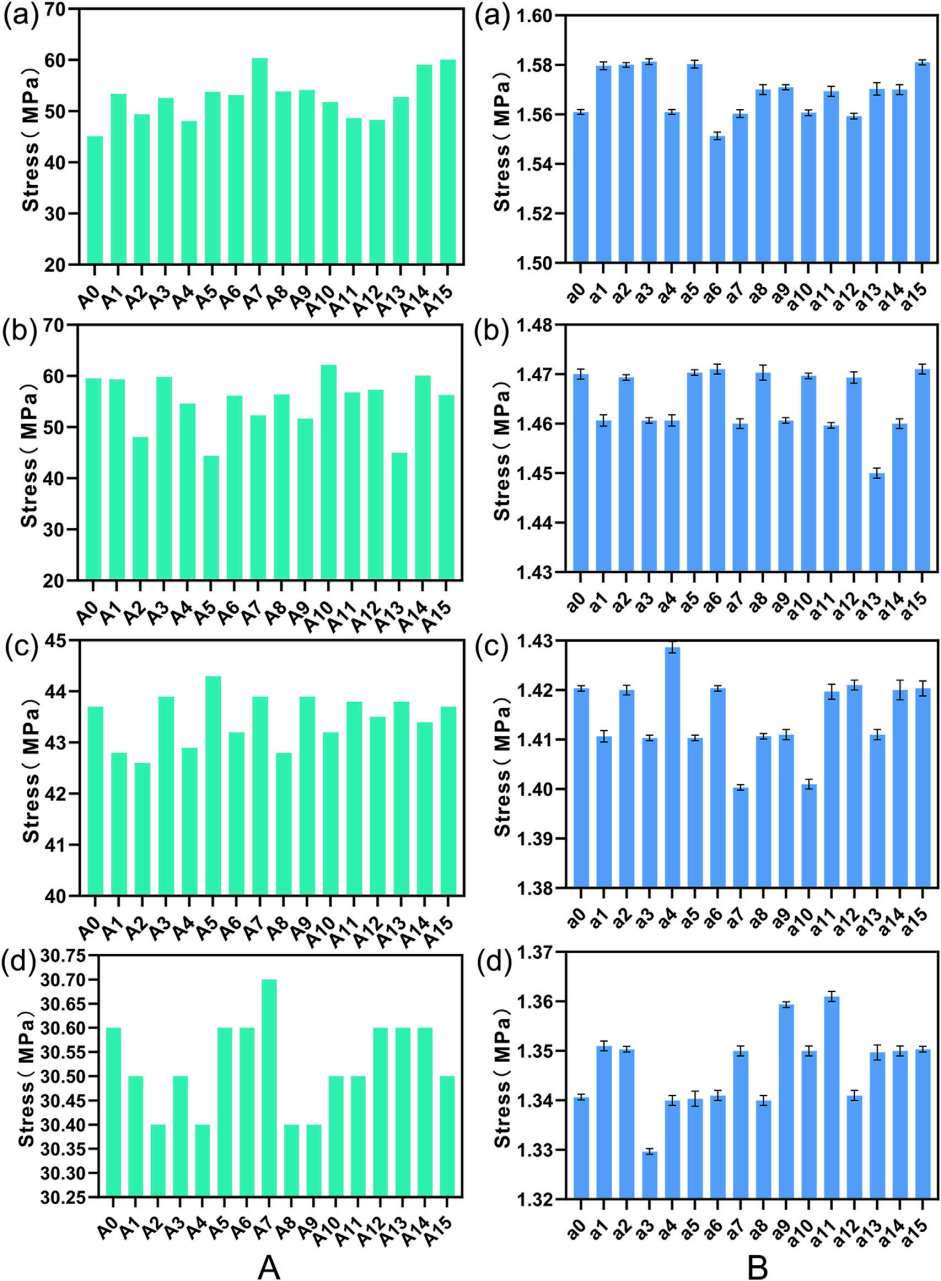
Stress on the L4 lower endplate under various conditions in **(A)** FE models and **(B)** calf lumbar spine models: **(A)** flexion, **(B)** extension, **(C)** lateral curvature, **(D)** torsion when the cage is positioned at different angles.

We plotted the stress nephograms of the L4 inferior endplate for the FE model with cage insertion angles ranging from 0°–16° (Figure 9). Figure 10 compares the stress on the L4 inferior endplate under flexion, extension, lateral bending, and torsion for 16 Stand-Alone OLIF FE models (A0-A15) and the corresponding calf lumbar spine models (a0-a15). Figure 10 indicates that in both the FE model and the *ex vivo* biomechanical experiment, there is no clear trend of change in endplate stress with the variation of the cage insertion angle under each condition, *p* > 0.05. The inclination angle of the cage has no significant effect on the stress experienced by the endplate after surgery.

### 3.4 Impact of the height of the cage

#### 3.4.1 Comparative analysis of intervertebral ROM

**FIGURE 11:**
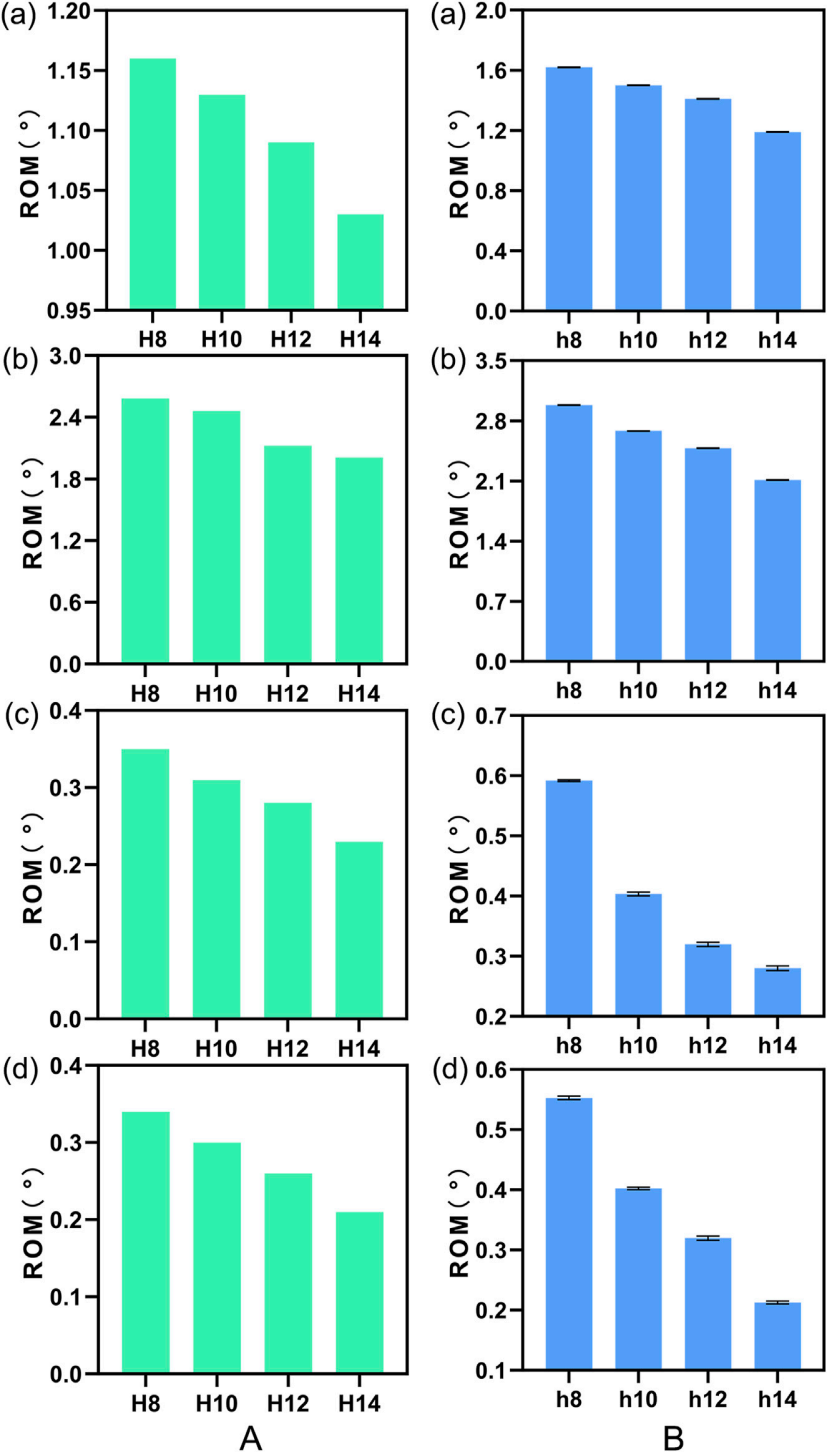
The ROM of **(A)** FE model L4-L5 segment and **(B)** calf lumbar spine model under various physiological activities: **(A)** flexion, **(B)** extension, **(C)** lateral curvature, **(D)** torsion when the cage is positioned at different heights.

Figure 11 illustrates a comparison of the Range of Motion (ROM) at the L4-5 segment under flexion, extension, lateral bending, and torsion for 4 Stand-Alone OLIF FE models (H1-H4) and the corresponding calf lumbar spine models (h1-h4). Figure 11A indicates that the range of motion in each direction of the L4-L5 segment in the FE model is less than that of the intact FE model. Comparing with Figure 4, it demonstrates that the stability of the lumbar spine is increased after Stand-Alone OLIF surgery. As the height of the cage increases, the intervertebral range of motion decreases under all conditions, leading to increased stability of the lumbar spine after surgery, *p* < 0.05. For Stand-Alone OLIF, selecting a cage with a greater height can enhance postoperative stability Figure 11B indicates that the trend of intervertebral range of motion changes with the height of the cage under various conditions in the calf lumbar spine model is consistent with that of the FE model, *p* < 0.05, which confirms the reliability of the FEA results. Under the same conditions, the *ex vivo* experimental range of motion is greater than the results of the FEA.

#### 3.4.2 Comparative analysis of endplate stress

**FIGURE 12:**
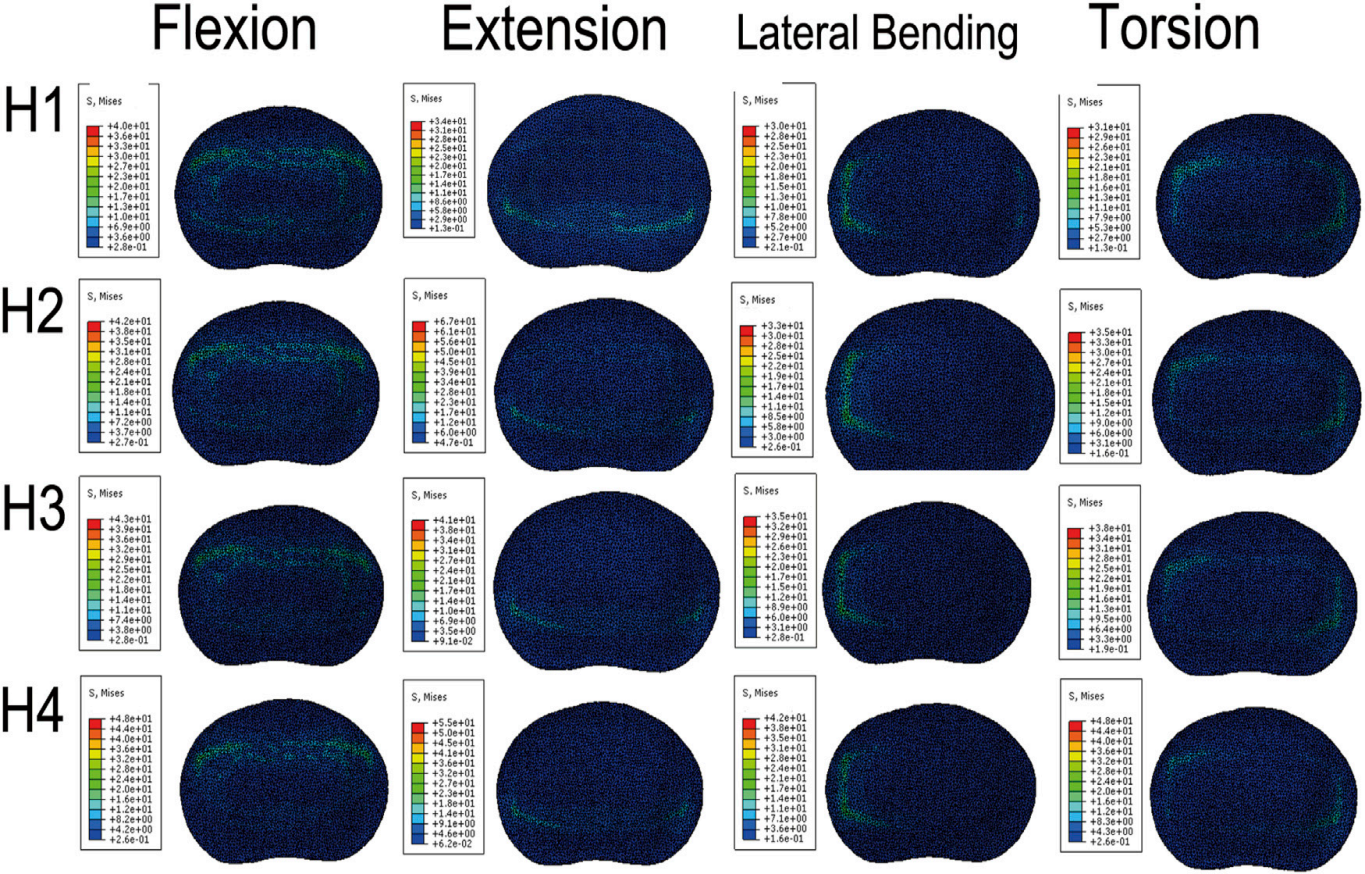
Stress cloud map of the L4 lower endplate in FE models with cages at 4 different heights under various conditions.

**FIGURE 13:**
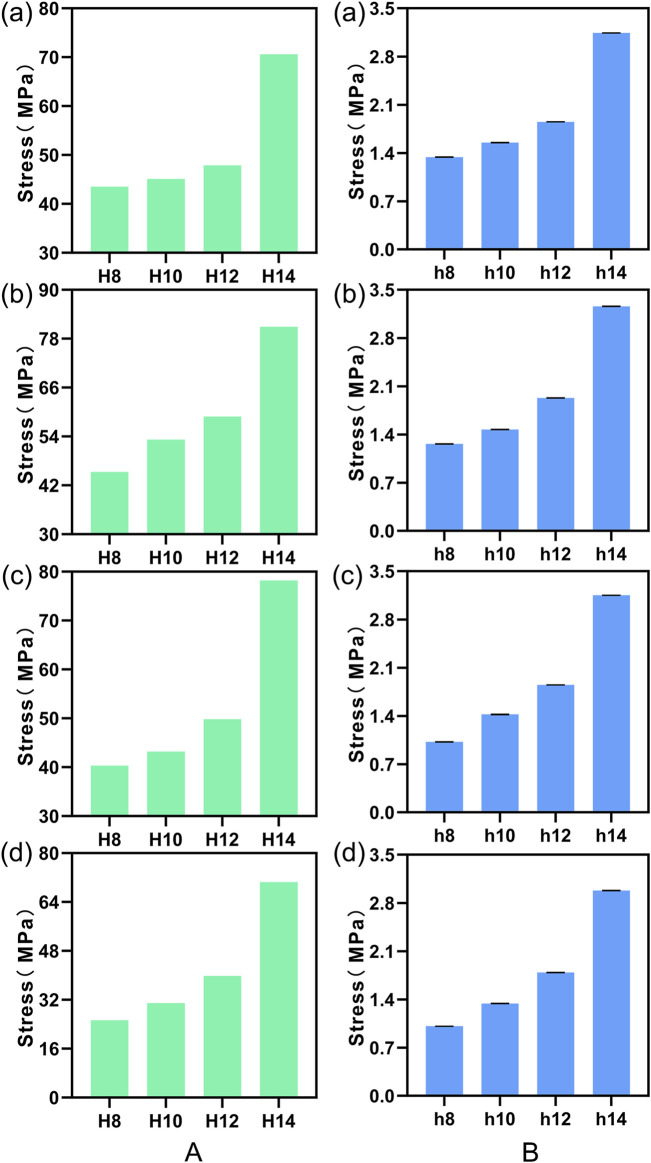
Stress on the L4 lower endplate under various conditions in **(A)** FE models and **(B)** calf lumbar spine models: **(A)** flexion, **(B)** extension, **(C)** lateral curvature, **(D)** torsion when the cage is positioned at different heights.

We plotted the stress nephograms of the L4 inferior endplate for the FE models with cages of heights 8mm, 10mm, 12mm, and 14 mm (Figure 12). Figure 13 illustrates a comparison of the stress on the L4 inferior endplate under flexion, extension, lateral bending, and torsion for four Stand-Alone OLIF FE models (H1-H4) and the corresponding calf lumbar spine models (h1-h4). Figure 13 indicates that in both the FE model and the *ex vivo* biomechanical experiment, the endplate stress under each condition increases with the increase in the height of the cage, *p* < 0.05. The endplate stress for the cage with a height of 14 mm is approximately twice that of the other three heights of cages. Within a certain range, the higher the height of the cage, the greater the endplate stress after Stand-Alone OLIF surgery. It is advisable not to choose a cage height exceeding 12 mm.

## 4 Discussion

The OLIF surgery, introduced only a little over a decade ago, has been increasingly favored by spinal surgeons due to its unique surgical approach and excellent clinical outcomes. The OLIF procedure, like ALIF and LLIF, avoids the destruction of the posterior spinal column structure associated with PLIF and TLIF, reducing neural and spinal cord complications (Phan et al., 2015; Barbagallo et al., 2014). At the same time, it addresses the limitations of ALIF and LLIF techniques, where anterior approaches may cause damage to iliac vessels and intra-abdominal structures (Rao et al., 2015), and lateral approaches are associated with injury to the psoas muscle and limitations in accessing the lower lumbar spine (Cawley et al., 2023; Taba and Williams, 2020). Due to the immediate stabilizing effect provided by larger cages, OLIF can achieve fusion without the need for additional internal fixation devices, thus avoiding the need for separate incisions and the potential for damage to the lumbar plexus and psoas muscle associated with the placement of screws or plates (Song and Wang, 2023). However, no lumbar fusion surgery technique is without drawbacks. The issue of endplate collapse after Stand-Alone OLIF surgery limits the widespread application of this technique.

This study focused on the biomechanical relationship between the cage and the endplate interface after Stand-Alone OLIF surgery, considering that endplate collapse may be related to excessive stress on the endplate after surgery and the surgical segment not reaching a stable state. Previous studies on spinal biomechanics mostly applied only one of FEA and *ex vivo* mechanical experiments. This study combined finite element technology with *ex vivo* mechanical experiments, and the results of both experiments corroborated each other, making the research conclusions more reliable. Additionally, this study aligned with actual clinical application scenarios. The placement position, inclination angle, and height selection of the cage were issues that surgeons needed to consider carefully. When considering the anterior-posterior placement of the cage, previous studies indicated that they divided the endplate into three or four regions (Qin et al., 2022; Nan et al., 2022), but in actual clinical practice, the positioning within each region still needs to be further refined. Therefore, this study refined the anterior-posterior position of the cage into 12 distinct segments. Similarly, regarding the inclination angle of the cage, since OLIF is a lateral oblique approach, the question of whether the implanted cage needed to be aligned straight had been addressed in previous studies, which typically categorized the cage’s orientation as neutral, oblique, or divided into increments of 5°–10° (Nan et al., 2022; Liang et al., 2020; Park et al., 2023), but in actual clinical practice further refinement is still needed. This study made full use of FEAs and *ex vivo* experiments, refining the angle to increments of 1°, and took into account the inclination angle of the cage from 0° to 15°. In the study of cage height, since the OLIF cages commonly used in clinical practice are 8mm, 10mm, 12mm, and 14mm, this study, adhering to clinical reality, analyzed the impact of these four heights of cages on endplate collapse following Stand-Alone OLIF surgery.

Lumbar fusion surgery provides stability to the lumbar spine by restricting the mobility of the targeted segment (Zhang et al., 2018). The results of this study showed that the Stand-Alone OLIF models with cages placed in 12 different positions at the L4-5 segment exhibited less mobility in all directions compared to the intact lumbar spine model. This proved that the placement of the cages enhanced the stability of the surgical segment following Stand-Alone OLIF surgery. The *ex vivo* mechanical testing results were consistent with the trends observed in the FEA. However, the impact of the cage placement position on the stability of the surgical segment varied to some extent. The position of the cage closer to the anterior edge of the endplate resulted in less mobility of the surgical segment during flexion and greater mobility during extension, which was generally consistent with the experimental results of Qin et al. (2022). From a biomechanical perspective, placing the cage at the anterior edge of the endplate provided stronger support to the front of the lumbar spine, restricting flexion movements while allowing extension movements. When the cage was placed too close to the anterior edge of the endplate, the mobility of the surgical segment significantly increased during lateral curvature and torsion, which indicated that the cage failed to provide lateral stability to the surgical segment at this position. The reason for this could be that the lumbar spine relied more on the stability of the middle and posterior columns during lateral curvature and torsion, while placing the cage too far forward may actually reduce the stability of the lumbar spine. After comprehensively considering the impact of cage placement on intervertebral mobility after Stand-Alone OLIF surgery, it was found that placing the cage in the center of the endplate achieved the optimal stabilizing effect. Furthermore, in this study, we discovered that in the Stand-Alone OLIF model, the cages with inclination angles from 0° to 15° all resulted in reduced mobility of the L4-5 segment in all directions compared to the intact lumbar spine model. This suggested that the placement of cages at these various angles could all contribute to improved stability of the lumbar spine following Stand-Alone OLIF surgery. The obliquity of the cage affected the stability of the surgical segment during different physiological activities in varying trends. During flexion and extension movements, the greater the obliquity of the cage, the less mobility there was in the surgical segment; during lateral curvature and torsion, the greater the obliquity of the cage, the more mobility there was in the surgical segment, which indicated that inserting the cage at an angle into the intervertebral space enhanced stability during flexion and extension but reduced stability in lateral curvature and torsion. By analyzing the causes, when the Stand-Alone OLIF cage had a certain inclination angle, it occupied a larger anterior-posterior space within the surgical segment’s intervertebral space, which was consistent with the direction of flexion and extension movements. This positioning allowed the cage to provide better support for the lumbar spine. However, the obliquity also reduced the lateral space occupied by the cage, resulting in decreased stability of the lumbar spine during lateral curvature and torsional movements. Consequently, the impact of the cage’s obliquity on the intervertebral activity after Stand-Alone OLIF surgery differed depending on the type of movement. After a comprehensive consideration, it was not possible to recommend an optimal obliquity as the best choice. At the same time, when we implanted cages of heights 8mm, 10mm, 12mm, and 14 mm into the Stand-Alone OLIF model, the mobility of the L4-5 segment in all directions was less than that of the intact lumbar spine model, which demonstrated that the insertion of cages at these four heights all enhanced the stability of the surgical segment after Stand-Alone OLIF surgery. The higher the height of the cage, the lower the mobility of the L4-5 segment postoperatively, which was consistent with the mechanical experimental results of the human lumbar spine by Du et al. (2017). This suggested that using a taller cage could lead to greater stability. Upon analysis, we found that the stability after Stand-Alone OLIF surgery depended on the supportive effect of the cage, and a taller cage offered more effective distraction of the intervertebral space. However, it was still not recommended to use cages above 12 mm for the following reasons: Firstly, previous studies have shown that excessive distraction of the intervertebral space is an important risk factor for adjacent segment disease (Kaito et al., 2011), and using a cage that is too tall may lead to the occurrence of adjacent segment disease. Secondly, the other three heights of cages also provide sufficient stability for Stand-Alone OLIF surgery.

The greater the stress on the endplate, the higher the risk of postoperative collapse (Kim et al., 2013). When the cage was positioned near the anterior edge of the endplate, the endplate experienced excessive stress during lumbar flexion; when it was positioned near the posterior edge, the endplate was subjected to high stress during lumbar extension. Lateral bending activities did not significantly alter the endplate stress regardless of the cage’s placement, but during torsional movements, the model with the cage centered showed lower endplate stress than those with off-center placements. This indicated that positioning the cage at the periphery of the endplate increased endplate stress, thereby raising the risk of postoperative endplate collapse. This discovery aligned with prior radiographic research. Kim et al. (2013) conducted follow-ups on 104 patients who had undergone TLIF surgery and observed that patients with cages positioned at the anterior edge of the endplate were more likely to have a higher incidence of cage subsidence postoperatively. Shiga et al. (2017) followed up with the postoperative radiographic data of 80 OLIF patients and found that endplate collapse was more frequent when the cage was placed closer to the anterior edge of the endplate. Our analysis of the impact of cage placement on endplate stress revealed two key points: Firstly, the anterior-posterior position of the cage affected the contact area between the cage and the endplate during lumbar motion. When the contact area was larger, the corresponding stress between the endplate and the fusion was smaller, with the largest contact area occurring when the cage is placed in the center of the endplate. Secondly, the position of the cage influenced the load line of the lumbar spine. Placing the cage in the center of the endplate aligned best with the original load line of the lumbar spine, resulting in the lowest stress on both the cage and the endplate. After comprehensively considering the effects of cage placement on endplate stress following Stand-Alone OLIF surgery, it has been determined that placing the cage at the center of the endplate resulted in the lowest likelihood of endplate collapse. The findings of this study demonstrated that in the Stand-Alone OLIF models with cages inserted at angles from 0° to 15°, the mobility of the L4-5 segment in all directions was reduced compared to that of an intact lumbar spine model. This indicated that the placement of cages at these various angles could all contribute to improved stability of the lumbar spine following Stand-Alone OLIF surgery. The obliquity of the cage had varying impacts on the stability of the surgical segment during different physiological activities. During flexion and extension, a larger inclination angle of the cage resulted in less mobility of the surgical segment. In contrast, during lateral curvature and torsion, a larger inclination angle of the cage resulted in greater mobility of the surgical segment. Our experimental results demonstrated that tilting the cage while inserting it into the intervertebral space enhanced stability during flexion and extension but compromised stability in lateral curvature and torsion. By analyzing the cause, when the Stand-Alone OLIF cage had a certain obliquity, it occupied a larger anterior-posterior space within the surgical segment’s intervertebral space, which was consistent with the direction of flexion and extension movements. This allowed the cage to provide better support for the lumbar spine. However, the obliquity also reduced the lateral space occupied by the cage, leading to decreased stability of the lumbar spine during lateral curvature and torsional movements. Therefore, the impact of the cage’s obliquity on intervertebral mobility after Stand-Alone OLIF surgery varied depending on the type of activities, and after comprehensive consideration, there was no single best tilt angle to recommend. Analyzing the stress on the L4 lower endplate after Stand-Alone OLIF surgery with four different heights of cages (8mm, 10mm, 12mm, and 14 mm), it was found that the higher the height of the cage, the greater the stress on the endplate postoperatively, and consequently, the higher the probability of endplate collapse. Particularly, the 14 mm cage significantly increased the postoperative endplate stress compared to the other three heights. Therefore, considering the impact of cage height on postoperative endplate stress in Stand-Alone OLIF surgery, it was advisable to choose a cage height that does not exceed 12 mm.

After analyzing the effects of cage placement on the mobility and endplate stress of the surgical segment after Stand-Alone OLIF surgery, considering flexion, extension, lateral bending, and torsion, it had been found that when the cage was placed in the center of the endplate, the stability of the lumbar spine was optimal and the stress on the L4 lower endplate was minimized. Correspondingly, the probability of postoperative endplate collapse was the lowest. As for the inclination angle of the cage, our study analyzed its impact on stability and endplate stress and found no influence on the risk of endplate collapse after Stand-Alone OLIF surgery. This was consistent with other radiographic follow-up results. Just as (Park et al., 2023) followed up with 118 OLIF surgery patients, categorizing the cage inclination angles as less than 10°, 10°–20°, and greater than 20°, and found no significant differences in radiographic outcomes. Liang et al. (2020) conducted a retrospective analysis of 98 patients who underwent lumbar interbody fusion surgery, dividing them into oblique placement and horizontal placement groups based on the angle of the cage. They compared the clinical and radiological outcomes before and after surgery for both groups and found that the clinical outcomes were the same for both groups. In our study examining the impact of cage height on the stability and endplate stress after Stand-Alone OLIF surgery, we found that a cage height not exceeding 12 mm is the optimal choice. Furthermore, a multitude of retrospective studies have established a strong correlation between the height of the interbody cage and the occurrence of postoperative endplate collapse. In their investigation into the risk factors for endplate collapse following OLIF surgery, Kotheeranurak et al. (2023) conducted follow-ups with 107 patients who received OLIF treatment, discovering that a taller cage height was most strongly associated with endplate collapse. Similarly, Satake et al. (2016), in their comparative analysis of the preoperative and postoperative X-rays of 102 patients who underwent LLIF surgery, identified a significant correlation between endplate damage and the height of the cage.

Undoubtedly, our current study still has some limitations. Firstly, our FE model did not simulate the paraspinal muscles, which may lead to discrepancies between our obtained data and actual situation. Secondly, because the three-dimensional structure of the ligaments cannot be simulated, we had to use one-dimensional spring materials as a substitute for the ligaments. Both of these situations limit the finite element analysis’s ability to accurately reflect reality. Although we conducted mechanical experiments on calf models, they still cannot perfectly reflect the conditions of the human lumbar spine. Thirdly, our comprehensive model was developed based on the geometric information of the lumbar spine obtained from a single individual. The geometric morphology of the lumbar spine varies from person to person, but to a certain extent, our model can only reflect the changes in the biomechanical trends of the lumbar spine under various loads. Nevertheless, our data is in good agreement with previous research and can reflect the impact of different cages on OLIF surgery appropriately.

## 5 Conclusion

For Stand-Alone OLIF surgery, inserting the cage in the central anterior-posterior position of the intervertebral space and selecting a cage with a height not exceeding 12 mm can reduce the stress on the endplate after surgery, which is more conducive to the stability of the lumbar spine postoperatively and reduces the risk of postoperative endplate collapse. The inclination angle of the cage placement does not significantly affect postoperative endplate stress or lumbar stability, and adjusting the inclination angle of the cage may not reduce the likelihood of postoperative endplate collapse.

## Data Availability

The original contributions presented in the study are included in the article/supplementary material, further inquiries can be directed to the corresponding author.
